# Assessment of the brain metabolome following innate immunity stimulation in a non‐human primate model of sporadic CAA

**DOI:** 10.1002/alz.090939

**Published:** 2025-01-09

**Authors:** Akash G Patel, Muhammad T Soliman, Lia Ficaro, Bharti P Nehete, Sohail Karimi, Fengzhu Wang, Hakyoung Kim, Lawrence E Williams, Pramod N Nehete, Drew R. Jones, Thomas Wisniewski, Henrieta Scholtzova

**Affiliations:** ^1^ NYU Grossman School of Medicine, New York, NY USA; ^2^ NYU Langone Health, New York, NY USA; ^3^ UT MD Anderson Cancer Center, Michale E. Keeling Center for Comparative Medicine and Research, Bastrop, TX USA

## Abstract

**Background:**

Non‐human primates (NHP) serve as an important bridge for testing therapeutic agents that have been previously shown to be effective in transgenic mouse models. Our earlier published data using an NHP model of sporadic AD‐related pathology that develops abundant cerebral amyloid angiopathy (CAA), squirrel monkeys (SQMs), indicates that chronic treatment with TLR9 agonist, class B CpG ODN, safely ameliorates CAA while promoting cognitive benefits. In the present study, we intended to delineate alterations in brain metabolome induced by chronic CpG ODN administration in order to provide further insight into CpG ODN immunomodulatory capabilities.

**Method:**

Global metabolomics analysis by HILIC‐LC‐MS was performed on frontal cortex tissue dissected from geriatric SQMs one month after the final CpG ODN or Vehicle (saline) injection. Tandem spectral (MS2) data was searched against the NIST/METLIN libraries for compound identification. Metabolite peak intensities were used for differential metabolite expression (p<0.05) and pathway analysis was performed using Metaboanalyst 5.0.

**Result:**

Differential metabolomics analyses revealed 151 putatively identified metabolites that were significantly altered in the brains of CpG ODN‐treated SQMs. Amino acids were the largest group of metabolites altered, with decreased levels in the CpG ODN group compared to the saline controls. ROC analyses identified several metabolites strongly associated with CpG ODN treatment, including amino acids such as proline and branched chain amino acids valine, leucine, and isoleucine. Furthermore, trimethylamine N‐oxide, a gut microbiome‐derived metabolite previously implicated in AD, was the most downregulated metabolite in the brains of CpG ODN monkeys relative to saline controls. Enrichment analysis identified significantly altered pathways including: “Aminoacyl‐tRNA biosynthesis”, “Valine, leucine and isoleucine biosynthesis”, and “Arginine biosynthesis.”

**Conclusion:**

Overall, the brain metabolome in an NHP model of naturally occurring CAA pathology was assessed for the first time. We observed decreased levels of several AD‐associated metabolites. These findings further develop our novel concept of immunomodulation in order to provide essential preclinical evidence for CpG ODN use as an effective drug for AD.

